# AhR transcriptional activity in serum of Inuits across Greenlandic districts

**DOI:** 10.1186/1476-069X-6-32

**Published:** 2007-10-23

**Authors:** Manhai Long, Bente Deutch, Eva C Bonefeld-Jorgensen

**Affiliations:** 1 Unit of Cellular and Molecular Toxicology, Department of Environmental and Occupational Medicine, Institute of Public Health, University of Aarhus, Denmark; 2 Centre for Arctic Environmental Medicine, Department of Environmental and Occupational Medicine, Institute of Public Health, University of Aarhus, Denmark

## Abstract

**Background:**

Human exposure to lipophilic persistent organic pollutants (POPs) including polychlorinated dibenzo-*p*-dioxins/furans (PCDDs/PCDFs), polychlorinated biphenyls (PCBs) and organochlorine pesticide is ubiquitous. The individual is exposed to a complex mixture of POPs being life-long beginning during critical developmental windows. Exposure to POPs elicits a number of species- and tissue-specific toxic responses, many of which involve the aryl hydrocarbon receptor (AhR). The aim of this study was to compare the actual level of integrated AhR transcriptional activity in the lipophilic serum fraction containing the actual POP mixture among Inuits from different districts in Greenland, and to evaluate whether the AhR transactivity is correlated to the bio-accumulated POPs and/or lifestyle factors.

**Methods:**

The study included 357 serum samples from the Greenlandic districts: Nuuk and Sisimiut (South West Coast), Qaanaaq (North Coast) and Tasiilaq (East Coast). The bio-accumulated serum POPs were extracted by ethanol: hexane and clean-up on Florisil columns. Effects of the serum extract on the AhR transactivity was determined using the Hepa 1.12cR mouse hepatoma cell line carrying an AhR-luciferase reporter gene, and the data was evaluated for possible association to the serum levels of 14 PCB congeners, 10 organochlorine pesticide residues and/or lifestyle factors.

**Results:**

In total 85% of the Inuit samples elicited agonistic AhR transactivity in a district dependent pattern. The median level of the AhR-TCDD equivalent (AhR-TEQ) of the separate genders was similar in the different districts. For the combined data the order of the median AhR-TEQ was Tasiilaq > Nuuk ≥ Sisimiut > Qaanaaq possibly being related to the different composition of POPs. In overall, the AhR transactivity was inversely correlated to the levels of sum POPs, age and/or intake of marine food.

**Conclusion:**

i) We observed that the proportion of dioxin like (DL) compounds in the POP mixture was the dominating factor affecting the level of serum AhR transcriptional activity even at very high level of non DL-PCBs; ii) The inverse association between the integrated serum AhR transactivity and sum of POPs might be explained by the higher level of compounds antagonizing the AhR function probably due to selective POP bioaccumulation in the food chain.

## 1. Background

Being resistant to both biotic and abiotic degradation, most persistent organic pollutants (POPs) bioaccumulate and magnify in animals and humans [1,2]. The polychlorinated dibenzo-*p*-dioxins/furans (PCDDs/PCDFs), polychlorinated biphenyls (PCBs) and organochlorine pesticides are dominating among the POPs. Some POPs e.g. PCBs and persistent organochlorine pesticides, that still are released into the environment due to ongoing use in developing countries and improper storage or disposal in developed countries, can be transported to the Arctic regions by atmospheric and oceanic currents and have been found to bioaccumulate and biomagnify in the Arctic marine food web [3,4]. The traditional diet of the indigenous people in the Arctic largely depends on fish, seabirds and marine mammals being associated with extraordinary high POP exposure [5-10], which may possess a health risk [11]. Since 1994, organochlorines have been measured in fat and plasma samples taken from Inuits of Greenland indicating that the contaminant level in Greenland is very high [5,8-10,12]. It has been documented that exposure to POPs such as dioxins (e.g. 2,3,7,8-tetrachlorodibenzo-*p*-dioxin, TCDD) and dioxin-like (DL) compounds including *non-ortho *and *mono-ortho *PCBs may cause a series of negative effects both in animals and humans including carcinogenicity [13], immunotoxicity and adverse effects on reproductive and neurobehaviour [14]. Most of the toxic and biological effects of dioxins and DL-compounds is mediated mainly through activation of the aryl hydrocarbon receptor (AhR), an intracellular ligand-dependent transcriptional factor expressed in most tissues of mammals [15]. Upon receptor-ligand binding and translocation into the nucleus, the complex with the AhR nuclear translocator (ARNT) binds to the DNA dioxin-responsive elements (DREs), causing induction of gene transcription, for instance, encoding for metabolic enzymes [16].

Since dioxins and DL-compounds exist as complex mixtures of various congeners in environmental and biologic samples, the concept of TEQ (TCDD toxic equivalent) has been introduced to simplify risk assessment and regulatory control [1,17]. The classical TEQs (WHO-TEQ) are calculated by multiplying the concentration of individual PCDDs/PCDFs/PCBs by their respective Toxic Equivalency Factors (TEFs), which correspond to the relative potency of the congener to generate AhR-mediated effects compared to TCDD, the most potent AhR ligand [1,17]. Studies have emphasized that assessment of the toxicological potential of a chemical mixture is much more complex than can be deduced by a calculated TEQ value [7,18,19]. There are several drawbacks using the TEF concept for risk assessment of mixtures of POPs such as expensive and time consuming gas chromatography mass spectrometry (GC-MS) determinations, small concentrations of hardly detectable individual congeners, presence of compounds not routinely measured or unknown substances with AhR affinity, the lack of TEF values for several POPs, and possible antagonistic or synergistic interactions between POPs [2,19-21]. Therefore, there is a need of simple, inexpensive and specific assays that can be used to give an integrated risk assessment of dioxins and DL-compounds. Rapid and sensitive AhR-based screening bioassays are valuable to more fully characterize the ligand-binding specificity of the AhR, to identify AhR ligands and AhR interfering compounds present in the extracts of environmental and biologic samples. A number of bioassays for the determination of TEQ values have been developed [22]. The experts have agreed that the use of *in vitro *bioassays provides an useful tool as a pre-screening method for TEQs in environmental samples [1,23]. The *in vitro *AhR mediated chemical activated luciferase gene expression (CALUX) bioassay is a reporter-gene based cell bioassay using genetically modified cells responding to AhR ligands. The CALUX bioassay has proven to be a quick and sensitive assay to detect the AhR mediated potential of pure chemicals [2,20,21,24], extracts of environmental and biological matrices eliciting the biological integrated net TEQ value (CALUX-TEQ) of complex mixtures as found in sediment, pore water [25], bovine and human milk [19,26], human serum [27] and follicular fluid [28]. Compared to the calculated chemical-derived TEQ (WHO-TEQ), the CALUX based AhR-TEQ might be more biological relevant for the risk assessment of dioxins and DL-compounds [29].

As a part of the human health program of the "Arctic Monitoring and Assessment Program (AMAP)"[30], the aim of the present study was i) to compare the AhR transcriptional activity of the actual mixture of lipophilic POP of serum of subjects from different districts in Greenland and ii) to evaluate whether the AhR transcriptional activity is associated to the profile and level of POPs and/or lifestyle factors.

## 2. Methods

### 2.1. Subjects, sampling and POPs determination

The subjects and sampling methods have been described in detail elsewhere [10]. Briefly, the participants were of Inuit descent from Nuuk, Sisimiut (South West Greenland), Qaanaaq (North West Greenland) and Tasiilaq (East Greenland). The men data of Sisimiut was also a part of the EU project INUENDO. Venous blood samples and questionnaires about demographic and lifestyle factors were collected. The serum was prepared as described [10] and then stored at -80°C until analyzed.

As described [10] plasma was analyzed for POPs including cis-chlordane, trans-chlordane, oxychlordane, *p,p'*-DDE, *p,p'*-DDT, hexachlorobenzene (HCB), beta-Hexachlorocyclohexane (β-HCH), mirex, toxaphene 26, toxaphene 50 and 14 PCB congeners (CB28, CB52, CB99, CB101, **CB105**, **CB118**, CB128, CB138, CB153, **CB156**, CB170, CB180, CB183, CB187; in bold are DL-PCB) by gas chromatography (GC). The samples enrolled per district were enough for comparison of the POP levels across districts with a power of 80% in power calculation. Plasma lipids were measured using conventional enzymatic methods and total lipids were calculated as described [31]. The lipid adjusted POP data (μg/kg lipid) was accomplished by dividing the wet-weight concentration in plasma (μg/l) by the individual samples lipid concentration and multiplying by 1000 to convert from gram to kilogram [10,31]. Because of high POP intercorrelation [10] (Bonefeld-Jorgensen and Long, submitted), the group-variables were used for evaluation of the data, namely, the sum of 14 PCB congeners (ΣPCB_14), the sum of pesticides (Σ pesticide) and finally the sum of all the determined POPs (Σ POP).

The fatty acid profiles were determined in plasma phospholipids at the Biology Department, University of Guelph, Canada [32]. The n-3 polyunsaturated fatty acids were reported on the sum of C18:3, n-3, C20:4, n-3, C20:5, n-3, C22:5, n-3 and C22:6, n-3, and the n-6 fatty acids was the sum of C18:2, n-6, C18:3, n6, C20:2, n-6, C20:3, n-6 and C20:4, n-6 [10]. The ratio between n-3 and n-6 is known to be a strong indicator of marine food intake and thus a good indicator of the relative consumption of traditional versus imported food [8,33].

### 2.2. AhR transcriptional activation assay

The serum was extracted with a mixture of ammonium sulphate/ethanol/hexane (1:1:3) and the lipid extract was concentrated and cleaned up on two Florisil columns to get the actual mixture of POPs [27,31]. The detailed descriptions of dissolving the extract samples and detecting serum AhR transcriptional activity has been described in detail elsewhere [27]. The stably transfected mouse hepatoma cell line Hepa1.12cR cells carrying the AhR-luciferase reporter gene was kindly provided by MS Denison (University of California, Davis, CA, USA). Similar with Ziccardi and co-workers [34], the optimal conditions in our assay for determination of AhR transcriptional activity of samples using Hepa1.12cR cell line were in 100 μl medium containing 10% FCS with 6 × 10^4 ^cells, final solvent concentration of 0.1–0.4% DMSO and 4 h of exposure. Further validation of our Hepa1.12cR bioassay was obtained by participating in two international programs of inter-laboratory comparisons of dioxin-like compounds (The second round of interlaboratory comparison of dioxin-like compounds in food using bioassays, 2004, Örebro University, Sweden, and Interlaboratory comparison of dioxins and dioxin-like compounds in food using CALUX bioassay, 2006, Scientific Institute of Public Health, Belgium). The limit of detection was 64 fg TEQ/well. The Hepa1.12cR cells were exposed for 4 hours to the serum extract alone (termed AhRag) or to mimic physiological environment in the presence of half maximum effective concentration of TCDD (termed AhRcomp) [27]. The luciferase activity was determined using a luminometer (BMG LUMISTAR) and cell protein was accomplished using a fluorometer (Wallac 1420 Multilabel Counter, Perkin Elmer life science, FIN) as previously described [35]. The luciferase activity of samples was presented as relative light units per microgram protein (RLU/μg protein) [35]. The average coefficient of variation (CV) between replicates (intra CV) and between day-to day independent assays (inter CV) of the solvent control was 9% and 17%, respectively and the intra CV of the test samples was 10%.

No cell toxicity on Hepa1.12cR cells was observed after exposure to the serum extract samples determined as described [35].

### 2.3. Calculation and statistical analysis

In the separate assays the activity differences between the triple serum extract determinations and their respective solvent controls [% agonist (samples with agonistic effect), % inhibition (samples with decrease of TCDD induced activity), and % increase (samples with further increase of TCDD induced activity)] were evaluated using the Student t-test with statistical significant level of p ≤ 0.05 (Microsoft Excel).

The AhR-TEQs values of serum extract were obtained by interpolation of the AhRag values onto the parallel TCDD dose-response sigmoid Hill curve and only the AhRag values being significantly higher than the solvent control and in the linear range of the TCDD dose-response curve were used to calculate AhR-TEQ [27].

Natural logarithmic transformation of AhR-TEQ/AhRcomp and POP data improved the normality (checked by Q-Q plots) and homogeneity of variance, and the statistical analysis was performed on the ln-transformed data. The comparisons of different variables (POPs, AhR-TEQ and AhRcomp) among the districts were performed with One-way ANOVA test. When ANOVA showed statistical significant differences complementary multiple comparison *ad hoc *tests was performed. Test for equal variances was performed using Levene's test. The least-significant difference (LSD) test was used if the variables showed equal variance; otherwise Dunett T3 test was used. The comparison of variables between the genders was performed by independent Student t-test.

Bivariate correlations were evaluated by Pearson correlation analyses.

The overall associations between the AhR-mediated activities and POPs across the study groups and/or across genders were assessed by comparing the regression lines for each study group/gender by using multiple regression analysis.

Up to date few studies on AhR transcriptional activity in human serum have been reported [27,36-39], and thus the knowledge is limited about which dietary or other lifestyle determinants might affect serum AhR transcriptional activity. Our hypothesis is that potential determinants of POP bioaccumulation might also be potential determinants for serum AhR transcriptional activity. Guided by the literature [40], age and seafood intake are known determinants affecting the POP serum level, and intake of seabird might as well influence the POP level. The n-3 fatty acids being determinants of seafood intake [8-10], and lifestyle factors such as smoking and BMI might also influence the serum POP level [8]. Using the multiple linear regression model, assessing the relation between the POPs and AhR activities, the impact of potential confounders were evaluated by entering variables together with ΣPCB_14, Σ pesticide and Σ POP as follows: in the first step, age and then seafood intake (represented by n-3/n-6 ratio) were included in the model, then additionally smoking years and BMI were included in the model. Finally, the bird intake was additionally included in the model. The potential confounder was defined if the regression coefficient (β) changes more than 10% and when a significant association (p ≤ 0.05) was obtained after adjustment. Then the calculations were adjusted for the confounders.

The inter-district variations in serum levels of POPs, AhR-TEQ and AhRcomp were also assessed by linear regression models and age was in these models considered as a potential confounder of these variables.

The statistical analysis was performed on SPSS 13.0 (SPSS Inc. Chicago, IL, USA). The statistical significant level was set to p = 0.05.

## 3. Results

### 3.1. The demographical and lifestyle factors of study groups

As reported [10], the age of the participants was from 18–77 with the median age of 35 years old. The Nuuk men were significantly older than the other participants, whereas the age range of the remaining male and female participants of all four districts were similar (Table 1). No significant difference was observed for the BMI of the participants from all districts. For the smoking years, Nuuk men and women had the highest median, whereas the other three districts were similar for both sexes (Table 1). The Nuuk and Sisimiut men had the highest and the lowest median n-3/n-6 ratio, respectively, whereas for women significantly higher n-3/n-6 ratio was observed for Qaanaaq and Tasiilaq. The median seabird intake of the Nuuk men was the highest whilst for the rest of subject's similar median seabird intake was observed (Table 1). Nuuk and Sisimiut participants had higher median consumption of dairy food than that of Qaanaaq and in overall male participants had lower dairy food intake than women (Table 1).

**Table 1:** Basic characteristics of study population

Men		Nuuk	Sisimiut	Qaanaaq	Tasiilaq	All
Age (years)	n	50	52	43	41	186
	median	54*	30	34	34	37
	mean	54	31	34	34	39
	min-max	35–77	18–46	19–45	19–45	18–77
BMI (kg/m^2^)	n	46	52	43	41	182
	median	28	26	27	26	26
	mean	28	27	27	26	27
	min-max	22–35	19–36	20–41	21–33	19–41
Smoking (years)	n	45	52	41	40	178
	median	34*	12	18	15	18
	mean	31	12	17	15	19
	min-max	0–59	0–26	0–31	0–31	0–59
n-3/n-6	n	50	37	43	41	171
	median	0.61*	0.22^▲^	0.40	0.40	0.40
	mean	0.74	0.29	0.48	0.45	0.50
	min-max	0.20–1.93	0.10–0.73	0.09–1.45	0.12–1.03	0.09–1.93
Seabird intake (per month)	n	47	49	41	na	137
	median	2.0*	1.0	1.0	na	2.0
	mean	5.5	2.6	2.6	na	3.6
	min-max	0–20	0–28	0–20	na	0–28
Dairy food consumption (per month)	n	48	51	43	na	142
	median	41	51	32*♠	na	41
	mean	39	49	30	na	40
	min-max	2–84	8–84	3–58	na	2–84
Women						
Age (years)	n	45	42	36	48	171
	median	38	31	34	29	34
	mean	36	33	33	30	33
	min-max	19–45	18–44	18–44	19–45	18–45
BMI (kg/m^2^)	n	43	42	36	48	169
	median	26	26	24	25	25
	mean	26	27	26	26	26
	min-max	18–32	19–47	18–34	17–40	17–47
Smoking (years)	n	42	42	34	48	166
	median	20	12	16	12	14
	mean	16	13	16	13	14
	min-max	0–33	0–27	0–29	0–31	0–33
n-3/n-6	n	45	38	36	48	167
	median	0.25	0.29	0.40^#^	0.41^#^	0.33
	mean	0.29	0.32	0.48	0.46	0.38
	min-max	0.12–0.72	0.14–0.82	0.15–1.53	0.18–1.0	0.12–1.53
Seabird intake (per month)	n	44	41	35	na	120
	median	1.0	1.0	1.0	na	1.0
	mean	1.4	1.3	3.4	na	1.9
	min-max	0–8.0	0–2.0	1.0–28	na	0–28
Dairy food consumption (per month)	n	45	42	35	na	122
	median	56	57	37	na	49
	mean	53	49	36	na	47
	min-max	2–84	0–84	0–84	na	0–84
Men + Women						
Age (years)	n	95	94	79	89	357
	median	44	31	34	32	35
	mean	46	31	33	32	36
	min-max	19–77	18–46	18–45	19–45	18–77
BMI (kg/m^2^)	n	89	94	79	89	351
	median	26	26	26	25	26
	mean	27	27	27	26	26
	min-max	18–35	19–47	18–41	17–40	17–47
Smoking (years)	n	87	94	75	88	344
	median	23	12	17	13	16
	mean	23	12	16	14	16
	min-max	0–59	0–27	0–31	0–31	0–59
n-3/n-6	n	95	75	79	89	338
	median	0.36	0.25	0.40	0.40	0.36
	mean	0.51	0.30	0.48	0.45	0.44
	min-max	0.12–1.93	0.10–0.82	0.09–1.53	0.12–1.03	0.09–1.93
Seabird intake (per month)	n	91	90	76	na	257
	median	2.0	1.0	1.0	na	1.0
	mean	3.5	2.0	3.0	na	2.8
	min-max	0–20	0–28	0–28	na	0–28
Dairy food consumption (per month)	n	93	93	78	na	264
	median	48	51	36	na	43
	mean	46	49	33	na	43
	min-max	2–84	0–84	0–84	na	0–84

na: not available.

*: p < 0.05 versus other districts; ^▲^: p < 0.05 versus Nuuk and Sisimiut men; ^#^: p < 0.05 versus Nuuk and Sisimiut women; ^♠^: p < 0.05 versus Nuuk and Sisimiut men and all women.

### 3.2. The plasma level of POPs

For the men, similar median levels of Σ PCB_14, Σ pesticide and Σ POP were observed among Nuuk, Qaanaaq and Tasiilaq and all were significantly higher than that of Sisimiut with the order of Nuuk ≥ Qaanaaq ≥ Tasiilaq > Sisimiut. Tasiilaq and Qaanaaq women had significantly higher POPs median levels than that of Nuuk and Sisimiut women (Table 2) as reported [10]. In general, male participants had higher median POP levels than women in all districts. For the combined sex data, Tasiilaq and Qaanaaq had similar POPs median levels which were significantly higher than that of Nuuk and Sisimiut participants (Tasiilaq ≥ Qaanaaq > Nuuk > Sisimiut). After adjustment for age, the pattern of regional difference did not change (data not shown).

**Table 2:** The plasma POP levels in the subjects of the different district

Men		Nuuk	Sisimiut	Qaanaaq	Tasiilaq	All
Σ PCB_14 (μg/kg lipid)	n	38	51	41	37	167
	median	2682	540.9*	1854	1890	1526
	mean	3094	752.1	2699	2079	2057
	min-max	490–11113	132–2366	617–7352	318–4527	132–11113
Σ pesticide (μg/kg lipid)	n	38	51	41	41	171
	median	3401	737.8*	2630	2242	2126
	mean	4034	1012	3824	2372	2684
	min-max	405–19118	141–3542	617–14366	194–7224	141–19118
Σ POP (μg/kg lipid)	n	38	51	41	37	167
	median	6322	1353*	4411	4067	3551
	mean	7127	1765	6524	4249	4692
	min-max	896–30231	285–5908	1234–19807	499–11260	285–30231
ΣDL-PCB/Σ PCB_14 (%)	n	38	51	41	37	167
	median	8.42^♠^	7.07	7.53	7.51	7.70
	mean	8.36	7.41	7.44	7.73	7.71
	min-max	5.0–11.0	4.0–11.0	4.0–11.0	6.0–11.0	4.0–11.0
Women						
Σ PCB_14 (μg/kg lipid)	n	44	42	34	47	167
	median	295.7	346	1188^▲^	1660^▲^	635.5
	mean	492.4	459.7	1406	1809	1041
	min-max	97.5–2428	67.2–1384	99–5980	112–6701	67.2–6701
Σ pesticide (μg/kg lipid)	n	44	42	34	48	168
	median	481.6	496.3	1671^▲^	2044^▲^	860
	mean	678	700	2018	2397	1446
	min-max	83.6–2881	75.6–3014	77.9–7529	153–6995	85.5–7529
Σ POP (μg/kg lipid)	n	44	42	34	47	167
	median	748	823	2847^▲^	3797^▲^	1469
	mean	1171	1159	3424	4169	2480
	min-max	181–5309	143–4337	177–12432	256–12829	143–12829
ΣDL-PCB/Σ PCB_14 (%)	n	44	42	34	47	167
	median	9.01	9.26	8.45	8.96	9.00
	mean	9.02	9.40	8.78	9.02	9.10
	min-max	4.0–13.0	6.0–15.0	6.0–13.0	6.0–12.0	4.0–15.0
Men + Women						
Σ PCB_14 (μg/kg lipid)	n	82	93	75	84	334
	median	912.4	495.3	1379^#^	1806^#^	1068
	mean	1698	620.0	2113	1928	1549
	min-max	97.5–11113	67.2–2366	99–7352	112–6701	67.2–11113
Σ pesticide (μg/kg lipid)	n	82	93	75	89	339
	median	1336	685.3	2156^#^	2208^#^	1327
	mean	2234	871	3006	2386	2071
	min-max	83.6–19118	75.6–3542	77.9–14366	153–7224	85.5–19118
Σ POP (μg/kg lipid)	n	82	93	75	84	334
	median	2322	1244	3437^#^	4020^#^	2420
	mean	3931	1491	5119	4206	3597
	min-max	181–30231	143–5908	177–19807	256–12829	143–30231
ΣDL-PCB/Σ PCB_14 (%)	n	82	93	75	84	334
	median	8.79	7.92	7.88	8.40	8.30
	mean	8.71	8.31	8.05	8.45	8.39
	min-max	4.0–13.0	4.0–15.0	4.0–13.0	6.0–12.0	4.0–15.0

Σ PCB_14: sum of 14 PCB congeners; Σ pesticide: sum of 10 pesticides; Σ POP: Σ PCB_14 + Σ pesticide; ΣDL-PCBs: sum of dioxin-like PCBs (PCB105, PCB118, PCB156).

*: p < 0.05, versus men from other districts; ^▲^: p < 0.05, versus Nuuk and Sisimiut women;

^#^: p < 0.05, versus Nuuk and Sisimiut subjects; ^♠^: p < 0.05, versus men from other districts.

The dioxin-like PCBs (DL-PCBs) determined in this study were CB105, CB118 and CB156. The Nuuk men had higher median percentage of ΣDL-PCB of the Σ PCB_14 than men from other districts (Nuuk > Tasiiaq ≥ Qaanaaq ≥ Sisimiut). No significant difference of ΣDL-PCB percentage between women from different districts was observed but a lower trend was observed for Qaanaaq women (Table 2). Women had significantly higher median percentage of DL-PCBs than men. In overall, the median percentage of ΣDL-PCB for the combined data was in the order of Nuuk ≥ Tasiilaq ≥ Sisimiut > Qaanaaq (Table 2).

### 3.3. AhR-TEQ and AhRcomp activity

**Figure 1:**
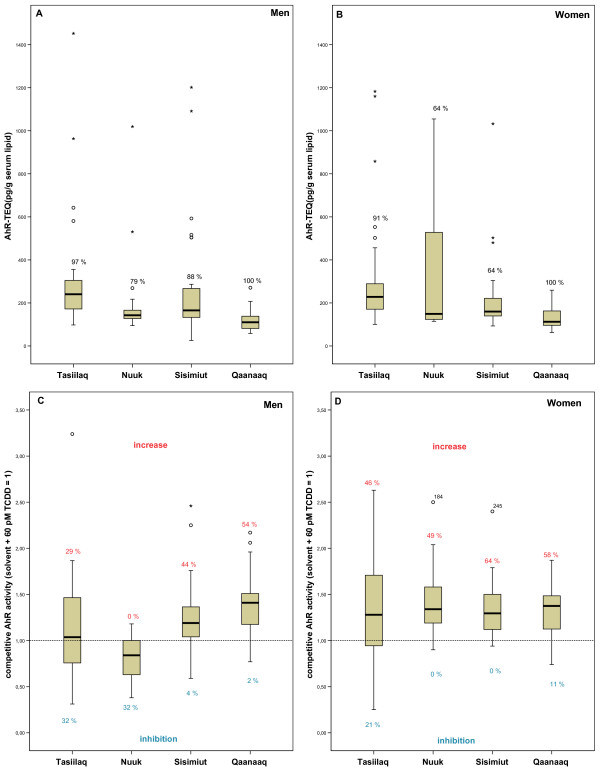
**The serum AhR transcriptional activity**. A) The AhR-TEQ in men from different districts; B) The AhR-TEQ in women from different districts; C) The AhRcomp activity in men from different districts and D) The AhRcomp activity in women from different districts. The reference line of the control is given as dash line (C, D). The values in the figure are percentage of sample with agonistic (A, B) and increase or inhibition of TCDD induced AhR transcriptional activity (C, D).

91% of all male and 79% of all female serum samples showed significant agonistic AhR transcriptional activity. The levels of AhR-TEQ differed significantly among the districts for the separate gender (Table 3, Fig. 1A &1B). The order of median level of AhR-TEQ was Tasiilaq ≥ Sisimiut ≥ Nuuk > Qaanaaq for men (Fig. 1A), and for women Nuuk ≥ Tasiilaq ≥ Sisimiut > Qaanaaq (Fig. 1B). Thus for both genders Qaanaaq had a lower AhR-TEQ level than participants from other districts (Table 3).

**Table 3:** The serum levels of AhR transcriptional activity of subjects of the different district

Men		Nuuk	Sisimiut	Qaanaaq	Tasiilaq	All
AhR-TEQ^1 ^(pg/g lipid)	n	21	42	38	38	139
	median	166	189	110*	240	153
	mean	206	247	118	296	219
	min-max	95 – 1019	26 – 1202	58 – 271	98 – 1452	26 – 1452
	% agonist	79	88	100	97	91
AhRcomp^2 ^(RLU/μg protein)	n	38	51	43	38	170
	median	0.78*	1.21	1.35	1.04	1.15
	mean	0.82	1.24	1.38	1.12	1.15
	min-max	0.38 – 1.18	0.59 – 2.46	0.77 – 2.17	0.31 – 3.24	0.31 – 3.24
	% increase	0	44	54	29	33
	% inhibition	32	4	2	32	16
Women						
AhR-TEQ^1 ^(pg/g lipid)	n	12	32	32	44	120
	median	235	188	122*	228	167
	mean	326	221	131	332	248
	min-max	115 – 1055	93 – 1032	63 – 259	100 – 1987	63 – 1987
	% agonist	64	64	100	91	79
AhRcomp^2 ^(RLU/μg protein)	n	45	42	36	44	167
	median	1.41	1.33	1.31	1.28	1.32
	mean	1.50	1.38	1.33	1.92	1.54
	min-max	0.9 – 4.54	0.94 – 3.88	0.74 – 1.87	0.25 – 15	0.25 – 15
	% increase	49	64	58	46	54
	% inhibition	0	0	11	21	8
Men + Women						
AhR-TEQ^1 ^(pg/g lipid)	n	33	74	70	82	259
	median	188	189	115	239	161
	mean	250	236	124	315	232
	min-max	94 – 1055	26 – 1202	58 – 271	98 – 1987	26 – 1987
	% agonist	71	76	100	94	85
AhRcomp^2 ^(RLU/μg protein)	n	83	93	79	82	337
	median	1.08	1.26	1.33	1.18	1.23
	mean	1.19	1.30	1.36	1.55	1.35
	min-max	0.38 – 4.54	0.59 – 3.88	0.74 – 2.17	0.25 – 15	0.25 – 15
	% increase	27	53	56	38	43
	% inhibition	15	2	6	26	12

^1^AhR-TEQ (TCDD equivalents): The AhR-TEQ of samples eliciting significantly agonistic activity was calculated by interpolation to the TCDD dose-response curve using the Sigmaplot program, given as pg/g serum lipid. The % agonistic indicates the % of samples eliciting a significant increase in AhR activity compared to the solvent control. ^2^AhRcomp: AhR competitive activity of serum extract + 60 pM TCDD (EC_50_) given as RLU/μg protein. EC_50 _solvent control = 1 RLU/μg protein; % increase and % inhibition indicates the % of samples responding with a further increase or decrease of the EC_50__(TCDD) _induced activity, respectively. *: p < 0.05 versus subjects from other districts.

AhRcomp activity was assessed by co-exposure with serum extract and 60 pM TCDD (EC_50(*TCDD*)_). The median AhRcomp activity of men was in the order of Qaanaaq ≥ Sisimiut ≥ Tasiilaq > Nuuk, reflecting the higher percentage of samples elicited inhibition of the TCDD induced AhR activity in the serum samples of Nuuk and Tasiilaq (Table 3, Fig. 1C). Higher percentage of serum samples of men from Qaanaaq and Sisimiut elicited a further increase of the TCDD induced AhR activity (Table 3, Fig. 1C). The serum samples of women from the four districts had similar AhRcomp level; mainly eliciting a further increase of the TCDD induced AhR activity (Table 3, Fig. 1D).

No significant difference of the AhR-TEQ level was found between the genders in the separate district (Table 3). In the Nuuk study group, men had a lower level of AhRcomp than women, whereas similar AhRcomp levels were found for the two genders for Sisimiut, Qaanaaq and Tasiilaq participants (Table 3).

Adjustment for age did not change the regional and gender difference of AhR-TEQ and AhRcomp (data not shown).

### 3.4. The association between AhR-TEQ, AhRcomp and POPs

#### 3.4.1. The correlations between the single POPs

Pearson correlation analyses showed that most PCB congeners were significantly and mutually intercorrelated (r > 0.82; p < 0.001), whereas CB 28, CB52, CB128 and CB101 had a relatively lower correlation coefficients to other PCB congeners (r = 0.29–0.82; p < 0.05). Also the determined pesticides were intercorrelated (r > 0.75 – 0.99). The grouping variables (ΣPCB_14 and Σ pesticide) intercorrelated with r = 0.97 and the sum of determined POPs as Σ POP was therefore acceptable.

#### 3.4.2. The correlations between AhR-TEQ, AhRcomp and POPs

##### 3.4.2.1. Multiple regressions of AhR-TEQ and AhRcomp on POPs across the study groups and genders

Multiple regression analysis of the combined districts and genders data was performed to analyze for homogeneity/heterogeneity of the associations between POPs and AhR-TEQ/AhRcomp (see Additional file 1). The analyses indicated that the associations of POPs and AhR-TEQ were allowed to be evaluated in each district for the combined gender, whereas the AhRcomp activity and POPs association must be evaluated in each single district for men and for women in combined districts.

However, in order to obtain the overall trend of the studied groups and a better statistical power, the data based on the combined genders of the single districts for POPs and AhRcomp and the combined genders across the districts were also evaluated for the correlation of POPs and AhR-TEQ/AhRcomp.

##### 3.4.2.2. Correlations between AhR-TEQ, AhRcomp and POPs

An inverse correlation between AhR-TEQ and POPs was observed for the combined men data and the total combined data (Table 4). Few significant correlations between AhR-TEQ and POPs were found for the single districts for the two sexes. Significantly inverse correlations between AhR-TEQ and the POP variables were observed for both the Qaanaaq women and the combined Qaanaaq participants before and after adjustment for age and/or n-3/n-6 (Table 4).

**Table 4:** Multivate linear regression analysis of AhR-TEQ and POPs

		Nuuk			Sisimiut			Qaanaaq			Tasiilaq			All		
Men^a^		n	β	p	n	β	p	n	β	p	n	β	p	n	β	p

*Σ PCB_14*	raw^1^	21	-.01	.97	41	-.24	.13	37	-.14	.41	33	-.06	.72	**135**	**-.21**	**.01**
	+age	21	.05	.86	41	-.09	.64	37	.05	.80	33	.03	.87	135	-.16	.11
	+n-3/n-6^2^	21	-.01	.98	29	-.25	.25	37	-.30	.23	33	-.11	.54	123	-.22	.07
	+age+n-3/n-6	21	.07	.87	29	-.06	.81	37	-.14	.60	33	.01	.98	123	-.16	.21
*Σ pesticide*	raw	21	.08	.72	41	-.27	.087	37	-.20	.23	37	-.13	.46	**139**	**-.25**	**<.01**
	+age	21	.15	.57	41	-.14	.44	37	-.01	.98	37	-.05	.78	**139**	**-.21**	**.04**
	+n-3/n-6^2^	21	.16	.61	29	-.34	.13	37	-.49	.06	37	-.20	.23	**127**	**-.37**	**<.01**
	+age+n-3/n-6	21	.22	.53	29	-.20	.39	37	-.30	.32	37	-.09	.60	**127**	**-.31**	**.01**
*Σ POP*	raw^1^	21	.04	.86	41	-.26	.10	37	-.19	.24	33	-.11	.56	**135**	**-.16**	**.01**
	+age	21	.11	.69	41	-.12	.50	37	-.01	.96	33	-.02	.93	135	-.20	.06
	+n-3/n-6^2^	21	.09	.78	29	-.30	.17	37	-.48	.07	33	-.15	.40	**123**	**-.29**	**.02**
	+age+n-3/n-6	21	.16	.66	29	-.15	.54	37	-.29	.31	33	-.04	.82	123	-.23	.06
Women^b^																
*Σ PCB_14*	raw^1^	12	.06	.85	32	.23	.20	**32**	**-.46**	**.01**	43	-.09	.55	119	-.04	.70
	+age	12	-.01	.99	32	.25	.24	**32**	**-.40**	**.05**	43	-.21	.35	119	-.002	.99
	+n-3/n-6^2^	12	.58	.19	28	.17	.43	32	-.32	.18	43	-.13	.50	115	.06	.62
	+age+n-3/n-6	12	.53	.31	28	.20	.38	32	-.25	.36	43	-.23	.35	115	.08	.53
*Σ pesticide*	raw^1^	12	.11	.74	32	.25	.16	**32**	**-.53**	**<.01**	44	-.11	.46	120	-.06	.52
	+age	12	.06	.89	32	.26	.19	**32**	**-.50**	**.01**	44	-.28	.20	120	-.03	.74
	+n-3/n-6^2^	12	.69	.12	28	.19	.39	**32**	**-.51**	**.04**	44	-.17	.35	116	.01	.97
	+age+n-3/n-6	12	.65	.20	28	.20	.37	32	-.47	.09	44	-.31	.19	116	.02	.89
*Σ POP*	raw^1^	12	.09	.79	32	.24	.18	**32**	**-.51**	**<.01**	43	-.11	.49	119	-.05	.57
	+age	12	.03	.95	32	.25	.22	**32**	**-.48**	**.02**	43	-.24	.28	119	-.02	.83
	+n-3/n-6^2^	12	.66	.14	28	.17	.42	32	-.47	.07	43	-.15	.43	115	.04	.77
	+age+n-3/n-6	12	.62	.23	28	.20	.39	32	-.42	.14	43	-.27	.27	115	.05	.67
Men + Women^c^																
*Σ PCB_14*	raw^1^	33	-.19	.29	74	-.05	.65	**70**	**-.32**	**.01**	77	-.10	.40	**254**	**-.15**	**.02**
	+age	33	-.09	.79	74	.03	.83	**70**	**-.22**	**.01**	77	-.07	.65	254	-.10	.18
	+n-3/n-6^2^	33	.12	.72	58	-.15	.30	**70**	**-.34**	**.02**	77	-.15	.24	238	-.16	.06
	+age+n-3/n-6	33	.24	.60	58	-.09	.53	70	-.26	.09	77	-.11	.49	238	-.12	.17
*Σ pesticide*	raw^1^	33	-.13	.47	74	-.07	.58	**70**	**-.38**	**<.01**	82	-.12	.28	**254**	**-.18**	**<.01**
	+age	33	.06	.84	74	.01	.94	**70**	**-.30**	**.03**	82	-.12	.38	254	-.13	.07
	+n-3/n-6^2^	33	.29	.37	58	-.16	.29	**70**	**-.47**	**<.01**	82	-.21	.09	**238**	**-.24**	**<.01**
	+age+n-3/n-6	33	.41	.29	58	-.12	.44	70	**-.40**	**.02**	82	-.17	.22	**238**	**-.21**	**.02**
*Σ POP*	raw^1^	33	-.16	.38	74	-.06	.59	**70**	**-.37**	**<.01**	77	-.12	.30	**254**	**-.17**	**.01**
	+age	33	-.001	.997	74	.01	.92	**70**	**-.29**	**.04**	77	-.10	.49	254	-.12	.09
	+n-3/n-6^2^	33	.23	.50	58	.16	.28	**70**	**-.45**	**<.01**	77	-.18	.17	**238**	**-.20**	**.02**
	+age+n-3/n-6	33	.36	.395	58	-.11	.46	70	**-.31**	**.02**	77	-.14	.36	238	-.16	.07

The analyses were performed on ln transformed data with AhR-TEQ as dependent and POPs as independent variables before and upon adjusted for the potential confounders.

^1^: non-adjusted data.

^2^: ratio of n-3 to n-6 fatty acids in serum.

^a^: Combining districts is not allowed according to the multiple regression analyses.

^b^: Combining districts is not allowed according to the multiple regression analyses.

^c^: According to the multiple regression analyses, combining sex for each district is allowed while combining sexes for all districts is not allowed.

For Σ PCB_14, Σ pesticide and Σ POP, AhR-TEQ, see the legend of Table 2 and Table 3

As shown in Table 5, the total combined data and the combined Tasiilaq data showed an inverse relationship between AhRcomp and POPs before and after adjustment for age and n-3/n-6. The significantly inverse correlations of AhRcomp and POPs observed for all men data disappeared upon adjustment for age and/or n-3/n-6 ratio (Table 5). Also for the combined Nuuk data, negative correlations of AhRcomp and POPs were observed, which disappeared upon adjustment for age plus n-3/n-6 ratio (Table 5). For the two sexes in the single districts, few significant correlations between AhRcomp and POPs were observed but Tasiilaq women showing an inverse correlation upon adjustment for age and/or n-3/n-6. In overall, negative association between AhRcomp and POP data was found but Sisimiut men where AhRcomp activity was positively related to the determined ΣPCB_14 upon adjustment for age and n-3/n-6.

**Table 5:** Multivate linear regression analysis of AhRcomp and POPs

		Nuuk			Sisimiut			Qaanaaq			Tasiilaq			All		
Men^a^		n	β	p	n	β	p	n	β	p	n	β	p	n	β	p

*Σ PCB_14*	raw^1^	38	-.11	.52	51	-.13	.36	41	-.07	.68	34	-.18	.31	**164**	**-.24**	**<.01**
	+age	38	-.16	.47	51	-.03	.87	41	-.001	1.00	34	-.29	.15	164	-.05	.56
	+n-3/n-6^2^	38	-.24	.37	37	.29	.12	41	.08	.68	34	-.20	.27	150	-.01	.95
	+age+n-3/n-6	38	-.30	.34	**37**	**.42**	**.04**	41	.11	.61	34	-.29	.15	150	.10	.35
*Σ pesticide*	raw^1^	38	-.09	.59	51	-.20	.17	41	-.12	.46	38	-.27	.11	**168**	**-.24**	**<.01**
	+age	38	-.10	.59	51	-.12	.45	41	-.07	.75	38	-.36	.05	168	-.06	.53
	+n-3/n-6^2^	38	-.16	.52	37	.22	.27	41	.01	.97	38	-.29	.10	154	-.04	.73
	+age+n-3/n-6	38	-.16	.52	37	.29	.17	41	.04	.87	38	-.36	.06	154	.07	.49
*Σ POP*	raw^1^	38	-.10	.55	51	-.18	.22	41	-.10	.53	34	-.19	.28	**164**	**-.24**	**<.01**
	+age	38	-.13	.53	51	-.09	.57	41	-.04	.84	34	-.30	.13	164	-.06	.53
	+n-3/n-6^2^	38	-.20	.43	37	.25	.20	41	.04	.86	34	-.21	.25	150	-.02	.87
	+age+n-3/n-6	38	-.22	.43	37	.34	.10	41	.07	.76	34	-.30	.13	150	.09	.46
Women^b^																
*Σ PCB_14*	raw^1^	44	.08	.60	42	17	.28	34	-.31	.07	43	-.18	.24	163	-.09	.24
	+age	44	.16	.37	42	.09	.59	34	-.15	.46	**43**	**-.51**	**.02**	163	-.11	.17
	+n-3/n-6^2^	44	.18	.25	38	.09	.62	34	-.49	.06	43	-.27	.14	159	-.11	.25
	+age+n-3/n-6	44	.25	.14	38	.07	.71	34	-.33	.25	**43**	**-.56**	**.02**	159	-.13	.21
*Σ pesticide*	raw^1^	44	.07	.67	42	.09	.57	34	-.25	.15	44	-.21	.18	164	-.11	.18
	+age	44	.11	.49	42	.-.001	.995	34	-.07	.72	**44**	**-.55**	**.01**	164	-.13	.13
	+n-3/n-6^2^	44	.18	.26	38	-.03	.85	34	-.37	.17	44	-.30	.10	160	-.14	.18
	+age+n-3/n-6	44	.22	.19	38	-.06	.76	34	-.19	.52	**44**	**-.59**	**.01**	160	-.15	.15
*Σ POP*	raw^1^	44	.08	.62	42	.12	.45	34	-.27	.12	43	-.195	.21	163	-.10	.21
	+age	44	.14	.42	42	.03	.85	34	-.10	.64	**43**	**-.54**	**.01**	163	-.12	.15
	+n-3/n-6^2^	44	.19	.24	38	.01	.97	34	-.42	.12	43	-.29	.12	159	-.13	.21
	+age+n-3/n-6	44	.24	.15	38	-.02	.94	34	-.24	.42	**43**	**-.59**	**.01**	159	-.14	.17
Men + Women^c^																
*Σ PCB_14*	raw^1^	**82**	**-.55**	**<.01**	93	-.05	.64	75	-.14	.22	77	-.22	.06	**327**	**-.24**	**<.01**
	+age	**82**	**-.35**	**.02**	93	-.07	.58	75	-.04	.76	**77**	**-.39**	**.01**	**327**	**-.19**	**<.01**
	+n-3/n-6^2^	**82**	**-.39**	**.01**	75	-.06	.66	75	-.07	.62	**77**	**-.27**	**.03**	**309**	**-.21**	**<.01**
	+age+n-3/n-6	82	-.21	.25	75	-.08	.55	75	-.001	1.00	**77**	**-.41**	**.01**	**309**	**-.18**	**.02**
*Σ pesticide*	raw^1^	**82**	**-.53**	**<.01**	93	-.10	.34	75	-.15	.20	**82**	**-.23**	**.04**	**332**	**-.24**	**<.01**
	+age	**82**	**-.30**	**.03**	93	-.13	.27	75	-.04	.80	**82**	**-.38**	**.01**	**332**	**-.18**	**<.01**
	+n-3/n-6^2^	**82**	**-.34**	**.02**	75	-.12	.35	75	-.08	.61	**82**	**-.28**	**.02**	**314**	**-.21**	**.01**
	+age+n-3/n-6	82	-.17	.28	75	-.14	.29	75	.01	.93	**82**	**-.40**	**<.01**	**314**	**-.18**	**.02**
*Σ POP*	raw^1^	**82**	**-.54**	**<.01**	93	-.09	.42	75	-.15	.21	**77**	**-.22**	**.05**	**327**	**-.24**	**<.01**
	+age	**82**	**-.32**	**.03**	93	-.11	.35	75	-.04	.80	**77**	**-.40**	**.02**	**327**	**-.19**	**<.01**
	+n-3/n-6^2^	**82**	**-.37**	**.01**	75	-.10	.43	75	-.07	.62	**77**	**-.27**	**.03**	**309**	**-.21**	**<.01**
	+age+n-3/n-6	82	-.19	.28	75	-.13	.35	75	.011	.95	**77**	**-.41**	**.01**	**309**	**-.18**	**.02**

The analyses were performed on ln transformed data with AhRcomp as dependent and POPs as independent variables before and upon adjusted for the potential confounders.

^1^: non-adjusted data.

^2^: ratio of n-3 to n-6 fatty acids in serum.

^a^: Combining districts is not allowed according to the multiple regression analyses.

^b^: Combining districts is allowed according to the multiple regression analyses.

^c^: Combining districts and sex is not allowed according to the multiple regression analyses.

For Σ PCB_14, Σ pesticide and Σ POP and AhRcomp, see the legend of Table 2 and Table 3.

Further adjustment for BMI, smoking year and bird intake did not change the pattern of associations of POPs and AhR-TEQ, AhRcomp (data not shown).

Scattered and few significant correlations of AhR-TEQ/AhRcomp and the single PCB and pesticides and/or Σ DL-PCBs were observed (data not shown). Since the congeners of PCBs were highly intercorrelated and the AhR-TEQ represents the integrated activity of the POP mixture, it is hard to assess which of the compounds having the highest impact on the AhR-TEQ/AhRcomp level.

### 3.5. Correlations between lifestyle factors, POPs and AhR transcriptional activity

#### 3.5.1. POPs

As found earlier [10], for the combined data the levels of ΣPCB_14, Σpesticide and ΣPOPs were positively associated with age (r > 0.449, p < 0.001) and seafood intake represented by n-3/n-6 ratio (r > 0.614, p < 0.001), and age was positively correlated to seafood intake (r = 0.472, p < 0.001).

#### 3.5.2. AhR-TEQ

As shown in Table 6, scattered and few correlations between AhR-TEQ and lifestyle factors were found in the single districts and the separate genders, most of which involved the age, and/or the n-3/n-6 ratio. A significantly negative correlation between age and AhR-TEQ was observed for Sisimiut men and the combined men data. For Sisimiut women, a positive correlation between BMI and AhR-TEQ and for Qaanaaq women a negative correlation of the n-3/n-6 ratio and AhR-TEQ were observed. Significantly negative correlation between age and AhR-TEQ was also found for the combined Qaanaaq data and the total combined data. For the combined Nuuk, Sisimiut and Qaanaaq data a significantly, positive correlation between AhR-TEQ and consumption of dairy food was found (Table 6), suggesting that dairy products may be one source of DL-compounds and/or AhR activating compounds for the Inuits living in West Greenland.

**Table 6:** The Pearson correlation coefficients of serum AhR-TEQ/AhRcomp and life style factors

	Men						Women						Men + Women					
	AhR-TEQ			AhRcomp			AhR-TEQ			AhRcomp			AhR-TEQ			AhRcomp		

*Nuuk*	n	r	p	n	r	p	n	r	p	n	r	p	n	r	p	n	r	p

age	20	-.08	.74	37	-.02	.91	11	.12	.72	44	-.13	.41	32	-.19	.28	**82**	**-.53**	**<.01**
n-3/n-6	20	-.01	.97	37	-.02	.91	11	-.32	.32	**44**	**-.30**	**.05**	32	-.26	.15	**82**	**-.50**	**<.01**
BMI	19	.40	.08	35	. 24	.15	11	.07	.84	42	-.19	.22	31	.13	.48	78	-.17	.14
smoking	19	.04	.86	32	.12	.52	8	.56	.15	34	.22	.21	27	.13	.53	66	-.22	.08
seabird	19	.001	1.0	34	.01	.94	8	-.47	.20	37	-.09	.56	28	-.19	.32	**72**	**-.43**	**<.01**
diary food	20	-.26	.25	36	-.12	.48	11	.34	.29	44	.014	.93	32	.03	.85	81	.19	.09
*Sisimiut*																		
age	**41**	**-.32**	**.04**	50	-.21	.14	31	.08	.65	41	.21	.18	73	-.18	.12	92	.01	.91
n-3/n-6	29	-.06	.75	36	-.31	.06	27	.21	.28	37	.29	.07	57	-.10	.48	74	.08	.55
BMI	41	-.05	.76	50	-.16	.28	**31**	**.52**	**<.01**	41	-.18	.27	73	.19	.10	92	-.16	.14
smoking	**35**	**-.36**	**.03**	43	-.12	.44	29	.11	.56	33	.01	.94	64	-.15	.22	76	-.03	.78
seabird	38	-.01	.97	46	-.12	.43	28	-.17	.38	38	-.05	.74	67	-.05	.72	85	-.12	.28
diary food	40	.15	.37	49	.06	.69	30	-.003	.99	40	.23	.15	71	.09	.44	90	.14	.18
*Qaanaaq*																		
age	37	-.28	.08	42	-.11	.48	31	-.33	.07	**35**	**-.34**	**.05**	**69**	**-.30**	**.01**	78	-.21	.66
n-3/n-6	37	-.002	.99	42	-.16	.30	**31**	**-.42**	**.02**	35	-.06	.74	69	-.17	.15	78	-.11	.31
BMI	37	-.02	.49	42	-.08	.60	31	-.11	.54	35	.04	.83	69	-.13	.28	78	-.02	.87
smoking	33	-.12	.49	37	-.12	.49	29	-.23	.22	**33**	**-.35**	**.05**	62	-.20	.12	70	-.21	.08
seabird	35	-.19	.27	**39**	**-.33**	**.04**	30	-.05	.81	34	-.23	.19	66	-.01	.37	74	-.28	.17
diary food	37	-.02	.91	42	.15	.34	29	-.01	.96	35	.14	.42	67	.001	.99	76	.14	.22
*Tasiilaq*																		
age	37	-.19	.25	37	.05	.76	43	.04	.81	43	.10	.53	81	-.07	.54	81	.03	.76
n-3/n-6	37	.26	.12	37	.01	.95	43	.12	.90	43	.02	.91	81	.12	.28	81	.007	.95
BMI	37	-.002	.91	37	-.002	.99	43	-.27	.08	43	-.08	.60	81	-.19	.10	81	-.07	.52
smoking	33	.01	.97	33	-.003	.99	41	.06	.72	41	.05	.75	74	.03	.79	74	.02	.90
seabird	na	na	na	na	na	na	na	na	na	na	na	na	na	na	na	na	na	na
diary food	na	na	na	na	na	na	na	na	na	na	na	na	na	na	na	na	na	na
*All*																		
age	**138**	**-.19**	**.03**	**169**	**-.35**	**<.01**	119	-.09	.34	166	.03	.66	**258**	**-.16**	**.01**	**336**	**-.20**	**<.01**
n-3/n-6	126	.10	.27	**155**	**-.20**	**.01**	115	-.12	.19	162	-.04	.59	242	-.02	.81	**318**	**-.16**	**.01**
BMI	137	-.02	.86	167	-.01	.77	119	.01	.87	164	-.02	.23	257	-.01	.93	332	-.09	.10
smoking	120	-.17	.07	**145**	**-.23**	**.01**	107	-.01	.96	141	.04	.68	227	-.11	.11	286	-.11	.06
seabird*	94	-.07	.47	**121**	**-.35**	**<.01**	68	-.20	.10	111	-.13	.16	163	-.12	.12	**233**	**-.33**	**<.01**
diary food*	99	.13	.18	129	.02	.79	72	.21	.08	119	.13	.15	**172**	**.17**	**.03**	249	.11	.08

na: not available

*: For the combined data of Nuuk, Sisimiut and Qaanaaq.

#### 3.5.3. AhRcomp activity

The AhRcomp data was also in overall inversely related to age and/or the n3/n6 ratio, but also the seabird intake seemed to be a determinant. A negative correlation for AhRcomp to seabird intake was observed for the Qaanaaq men and all men data (Table 6). Moreover, the AhRcomp of all men was found to be inversely correlated to age, n-3/n-6 ratio and smoking years. Negative correlations between AhRcomp and the n-3/n-6 ratio and age and smoking years were found for the women of Nuuk and Qaanaaq, respectively. For the combined sex data of Nuuk subjects, negative correlations between AhRcomp and age, n-3/n-6 ratio and seabird intake were found (Table 6). By combining all data (districts and sexes), AhRcomp was found to be negatively correlated to age and n3/n6 ratio (Table 6). Besides, seabird intake was found to be negatively correlated to AhRcomp for all men data, the total combined data of Nuuk, Sisimiut and Qaanaaq groups (Table 6).

## 4. Discussion

In the present study we compared the *ex vivo *net integrated AhR transcriptional activity of the lipophilic serum POP mixture of Inuits among different Greenlandic districts and evaluated whether the serum AhR transcriptional activity was related to the bioaccumulated POPs and/or lifestyle factors. The serum levels of POPs, AhR-TEQ and AhRcomp activity differed among the two sexes living in different Greenlandic districts (Northwest-Qaanaaq, South West-Nuuk and Sisimiut and East-Tasiilaq). Also the association of the AhR transcriptional activity to the determined POPs differed between the districts and genders. We observed for the combined data an inverse association between AhR-TEQ and POPs where the Qaanaaq women had main influence. The negative association of AhRcomp activity to POPs was mainly contributed by Nuuk and Tasiilaq subjects.

As reported [10], the POPs levels of Tasiilaq and Qaanaaq participants (including men and women) were in general higher than that of Nuuk and Sisimiut subjects, which is consistent with previous reports that inhabitants of East Greenland had higher POP levels than those in the West Greenland [6,8,41] and in accordance with POP levels in the marine biota of North West Greenland and East Greenland being higher than that of the Central West Coast [41]. A greater prevalence of western lifestyle habits (consumption of imported food) was reported for Nuuk and Sisimiut subjects [10,12], which can explain the lower POP burden in Sisimiut and Nuuk subjects compared to Qaanaaq and Tasiilaq. However, in the present study, Nuuk men elicited higher POP levels than men from other districts, which may be due to their higher age and high intake of seafood and seabird [10,42].

The pattern of regional differences of serum AhR-TEQ levels did not follow the district-distribution of POPs levels. The AhR-TEQ level of Nuuk men having the highest POP level was lower than that of Sisimiut men with the lowest POPs level, whereas Qaanaaq women with relatively higher POPs level elicited the lowest AhR-TEQ level. However, the regional differences of AhR-TEQ seemed to mimic the district variation of the ratio of the ΣDL-PCB to the ΣPCB_14. For both sexes, the DL-PCB proportion of Qaanaaq subjects was in general lower than that of Nuuk, Tasiilaq and Sisimiut (men: Nuuk > Tasiilaq ≥ Qaanaaq ≥ Sisimiut; women: Sisimiut ≥ Nuuk ≥ Tasiilaq ≥ Qaanaaq) and consistently lower AhR-TEQ level was observed for Qaanaaq men and women (men: Tasiilaq ≥ Sisimiut ≥ Nuuk > Qaanaaq; women: Nuuk ≥ Tasiilaq ≥ Sisimiut > Qaanaaq). Similarly, male participants in all districts had higher POP burden but lower DL-PCB proportion and accordingly a tendency of lower AhR-TEQ level compared to female was observed in the present study. Moreover, we also found that individuals with higher ΣDL-PCB/Σ PCB_14 ratio tend to have higher AhR-TEQ levels while those with higher Σ POP have significantly lower AhR-TEQ than individuals with lower Σ POP (data not shown). Our study further support the previous report that DL-PCBs were major contributors of the total WHO-TEQ concentration in the plasma of Arctic Inuits [43]. Therefore, the total POPs level may not be a suitable proxy of the DL-compounds levels and thus the composition of the POPs in the body must be taken into account for the assessment of the net AhR transcriptional activity of serum samples [27].

Ayotte and coworkers reported that the average plasma WHO-TEQ level of Inuit adults living in Arctic Quebec was 184 pg/g lipid [43], being compatible with the serum AhR-TEQ level of Greenlandic Inuits determined in the present study (161 pg/g lipid). Previously, we reported a lower AhR-TEQ level for Greenlandic Inuit males (197 pg/g lipid) than for European men from Sweden, Warsaw and Kharkiv with the average serum AhR-TEQ level of 317 pg/g lipid [27]. The present study further supports that Greenlandic Inuits have lower level of AhR-TEQ than European individuals. The lower level of AhR-TEQ in Inuits compared to Europeans might be due to lower level of DL-compounds and lipophilic AhR activating compounds as well as higher level of AhR inhibiting POPs in the Inuit samples in addition to the report of lower level of PCDD/Fs in the Arctic region versus Europe and America [44]. Furthermore, re-evaluation of our previous study [27] by stratifying on the Inuits and European Caucasians showed a significantly positive correlation of POP proxy marker CB-153 and AhR-TEQ in European Caucasians (r_s _= 0.20, p = 0.001), combined with their higher AhR-TEQ level, suggesting European Caucasians were more close to DL-compounds sources.

Knowing that the measured net integrated AhR induced activity of the lipophilic serum extract are in the linear range (Bonefeld-Jorgensen and Long, submitted), and running a TCDD dose-response measurement in parallel, we suggest that the chemical mixture in serum extract can further increase or inhibit the TCDD induced AhR mediated activity (termed as AhRcomp). In the present study, the AhRcomp differed between districts and between sexes. Compared to Qaanaaq and Sisimiut men, Nuuk and Tasiilaq men had the lowest AhRcomp median level being in accordance with the higher frequency of samples eliciting inhibition of the TCDD induced AhR activity, suggesting the presence of compounds having the potency to compete with TCDD for the AhR activation. The higher incidence of further increase of the TCDD induced effect observed for Sisimiut and Qaanaaq men as well as women from all districts suggest the presence of compounds interacting with TCDD for AhR mediated effects. The differences in the incidence of further increase and inhibition of TCDD induced activity might be caused by the different POP composition of Inuits from the different districts and for genders also differences in diet intake might have an impact on the AhR function [8,10].

Humans are exposed to a combination of several xenobiotics. It is likely that the interaction between these contaminants, additivity and/or synergy and/or antagonism, will be reflected in the final toxic effect. *In vivo *and *in vitro *studies have reported that PCB mixtures and some individual PCB congeners antagonized TCDD effects [45,46], and Arochlor 1254 was reported to inhibit TCDD induced 7-ethoxyresorufin O-deethylase (EROD) activity [47]. A mixture of PCBs, based on the congeners identified in human milk, significantly decreased TCDD induced rat liver EROD activity [45]. For the POPs determined in the serum extract in this study, only 3 DL-PCBs (CB105, CB-118 and CB156) were shown to have agonistic AhR potentials [1,17,23], while other PCBs such as PCB153, PCB 52, PCB128 have the potential to antagonize the AhR pathway [20,48] and HCB was also reported to inhibit the TCDD induced activity [49]. Chlordane was reported to decrease the basal and TCDD induced CYP 1A1 promoter activity in MCF-7 cells [50]. Furthermore, most of the determined pesticides and PCBs in this study were reported as xenoestrogens (p,p'-DDT, p,p'-DDE, toxaphene, chlordane) [51-54] or antiestrogens (CB138, CB153, CB180) [55,56]. Previous studies showed that o,p'-DDT and other xenoestrogenic pesticides such as endosulfan and dieldrin significantly decreased the basal and TCDD induced AhR mediated gene expression [50,57]. Moreover, high concentrations of weak agonists can lead to antagonism [21,58]. Also, there may be other forms of antagonism when there are large concentrations of other compounds relative to high affinity ligands. Non-DL-PCBs can both antagonize and/or synergize the effects of DL-compounds, it depends on the response, the dose, and the concentrations [48,59]. As humans are exposed to a complex mixture and many PCBs can act as AhR antagonists most likely in concert when present together, this could actually influence the overall effect of DL-compounds by decreasing the net serum AhR transcriptional activity. Since the serum AhR transcriptional activity determined in the present study is the net integrated effect of the AhR agonists and antagonists of the constituents in the mixture, the negative correlation of AhR-TEQ, AhRcomp and POPs/PCBs/pesticides observed might be due to a higher concentration of compounds having antagonistic activity on AhR transactivity.

The biomagnification of PCBs in the aquatic food-chain is congener-specific. For most marine mammals, non-DL-PCBs are observed more resistant to biodegradation than DL-PCBs [60] and a previous study reported that the concentration of CB77, CB126 and CB169 showed no obvious biomagnification although the concentration of non-DL-PCBs such as CB138 increased from plankton to piscivores to herring gulls [61]. The lack of biomagnification of CB77 was attributed to its rapid elimination by aquatic species [62] and the CB105 and CB118 are more easily metabolized congeners than non-DL-PCBs such as CB153 [63]. Owing to this selective biotransformation during the bioaccumulation in the food chain [10], the concentration of some DL-PCBs might be reduced, whereas non-DL-PCBs accumulate, causing the variation of the composition of bioaccumulated PCBs.

In overall, the serum AhR-TEQ/AhRcomp negatively correlated to age and n-3/n-6 ratio, being in accordance to the frequency of marine food intake and POPs bioaccumulation over time. For the separate districts and genders a scattered correlation of AhR-TEQ/AhRcomp and lifestyle factors was observed. This non-consistent pattern might be explained by relative low statistical power for the separate gender and/or districts and needs further confirmation.

The inverse correlation of AhR-TEQ to seafood intake given by the n-3/n-6 ratio, suggests that the AhR transcriptional activity might follow the intake of n-6 fatty acids possibly with the origin from non-marine diet being supported by our previous report (Bonefeld-Jorgensen and Long, submitted). Moreover, it was reported that the important source of DL-compounds for the population of the western world is dairy food intake [64-66]. In the present study, we observed that the consumption of dairy food was higher for the Nuuk and Sisimiut participants than that of Qaanaaq and a positive correlation between dairy food intake and serum AhR-TEQ level was observed for the combined Nuuk and Sisimiut data and the combined Nuuk, Sisimiut and Qaanaaq data, respectively. Being more westernized, the import of western food and intake of milk products was reported to be higher for the subjects living in Nuuk and Sisimiut, while the intake of local Greenlandic products was relatively higher in Qaanaaq [10]. Hence, the dairy food might be an important source of DL-compounds for the Inuits living in bigger cities such as Nuuk and Sisimiut, relating to the higher AhR-TEQ level observed for Nuuk and Sisimiut compared to Qaanaaq. Moreover, we observed that POPs increase significantly with age for Inuits in this study as reported [10] as well as for Inuits and Europeans in an earlier study [67]. Evaluation of our previous data [27] showed that in Europeans the AhR-TEQ positively correlated to age (r = 0.12, p = 0.05), whereas for Inuits negative correlation was observed (r = -0.3, p = 0.01), suggesting different POP profiles resulted in different outcome on the AhR transcriptional activity.

Although the correlations found in the present study were weak, the chance finding may be excluded because the significant correlations were higher than 5%.

In the present study, the AhR transcriptional activation of serum extract was measured as an integrated net AhR-TEQ value without providing information on the levels of individual congeners. This bioassay responds to environmental contaminants acting through the AhR-pathway and allows the detection of chemicals that are not included in the chemical analytical method based on the standard TEF scheme. However, as discussed previously [27], upon serum extraction and clean-up the polycyclic aromatic hydrocarbons (PAHs) and other plant dietary AhR agonists are not expected to contribute to our determined AhR transcriptional activity (personal communication, Pierre Dumas, Quebec, Canada). The CALUX bioassay including AhR transcription activation assay takes into account all possible interactions (synergistic, additive and/or antagonistic interaction) between congeners while chemical analytical method like GC-MS focuses on a selected numbers of compounds (i.e. those documented to produce AhR-dependent toxicity) and only assumes the additive interaction of known detectable compounds. Therefore, the AhR-TEQ value derived from CALUX bioassays (AhR transcription activation bioassay) and chemical methods (WHO-TEQ) can not be expected to be equal. In most of the cases, higher AhR-TEQ value are usually obtained with AhR-CALUX bioassays compared to the calculated WHO-TEQ derived from chemical method [18,68]. However, the AhR-TEQ and WHO-TEQ values derived from AhR transcriptional activation CALUX bioassays and chemical analyses, respectively, are correlated [68] and these two methods are complimentary where the AhR transcription activation CALUX bioassay provides an overall biological response of the mixture, whereas the chemical analysis provides the concentration of specific compounds. Thus the key application of AhR transcriptional activation CALUX bioassay is screening and prioritization of samples [28] and is a valuable tool for comparison of different level of AhR transcriptional activity including DL-activity across various population.

## 5. Conclusion

The POPs and serum AhR transcriptional activity differed among Greenlandic districts, suggesting that the difference of serum AhR transcriptional activity depends on the composition of bioaccumulated POPs. The observed inverse correlation of POPs and AhR-TEQ or AhRcomp data suggests the presence of compounds with antagonistic impact on the AhR signalling pathway, probably due to the selective PCB bioaccumulation in the food chain. Further study is needed to elucidate this preliminary conclusion.

## Abbreviations

**AhR **aryl hydrocarbon receptor

**AhRag **agonistic AhR activity

**AhRcomp **competitive AhR activity

**CALUX **Chemical activated luciferase gene expression

**DDT **2, 2-bis (p-chlorophenyl)-1, 1, 1-trichloroethane

**DL **dioxin-like

**GC-MS **gas chromatography – mass spectrometry

**HCB **hexachlorobenzene

**β-HCH **Hexachlorocyclohexane

**PAHs **polycyclic aromatic hydrocarbons

**PCDDs/PCDFs **polychlorinated dibenzo-p-dioxins/furans

**PCBs **polychlorinated biphenyls

**POPs **persistent organic pollutants

**TCDD **2,3,7,8-tetrachlorodibenzo-p-dioxin

**TEFs **Toxic Equivalency Factors

**TEQs **TCDD toxic equivalents

## Competing interests

The author(s) declare that they have no competing interests.

## Authors' contributions

ML and EBJ were responsible for design of the AhR transcriptional activity study and preparation of the manuscript; ML performed the mechanistic work and data evaluation. BD has provided the epidemiological data concerning POPs, questionnaires and fatty acids. All authors have read and approved the final manuscript.

## Supplementary Material

Additional file 1 Multiple regressions of the combined study groups. The data provided represents the statistical analysis of the homogeneity/heterogeneity of the associations between POPs and AhR-TEQ/AhRcomp among the districts and genders. Click here for file
