# Supplementary material for: AhR transcriptional activity in serum of Inuits across Greenlandic districts
Source: Environ Health. 2007 Oct 23;6:32. doi: 10.1186/1476-069X-6-32 (PMC2173889; doi:10.1186/1476-069X-6-32)
Supplement: Additional file 1 — Multiple regressions of the combined study groups. The data provided represents the statistical analysis of the homogeneity/heterogeneity of the associations between POPs and AhR-TEQ/AhRcomp among the districts and genders. [file 1476-069X-6-32-S1.pdf]

Supplementary Table. Multiple regressions of the combined study groups

Across districts (men + women)				
Response variable	Homogeneity of slope (p value)	Common slope, Estimate (SE), p value	Common intercept (p value)	R^2
**AhR-TEQ (n=254)**				
∑ PCB_14	0.80	**-0.09 (0.04), 0.03**	**<0.001**	0.22
∑ pesticide	0.73	**-0.09 (0.04), 0.02**	**<0.001**	0.23
∑ POP	0.74	**-0.1 (0.04), 0.02**	**<0.001**	0.22
**AhRcomp (n=327)**				
∑ PCB_14	**0.01**	-*	-*	-*
∑ pesticide	**0.02**	-*	-*	-*
∑ POP	**0.02**	-*	-*	-*
**Across districts (men only)**				
**AhR-TEQ (n=135)**				
∑ PCB_14	0.71	-0.12 (0.07), 0.10	**<0.001**	0.21
∑ pesticide	0.45	-0.11 (0.06), 0.07	**<0.001**	0.22
∑ POP	0.56	-0.12 (0.07), 0.07	**<0.001**	0.21
**AhRcomp (n=164)**				
∑ PCB_14	0.77	-0.06 (0.04),0.13	**<0.001**	0.30
∑ pesticide	0.45	**-.007 (0.03), 0.03**	**<0.001**	0.29
∑ POP	0.77	-.006 (0.04), 0.08	**<0.001**	0.30
**Across districts (women only)**				
**AhR-TEQ (n=119)**				
∑ PCB_14	0.22	-0.03 (0.06), 0.61	**<0.001**	0.19
∑ pesticide	0.11	-0.04 (0.06), 0.54	**<0.001**	0.20
∑ POP	0.14	-0.04 (0.06), 0.54	**<0.001**	-0.19**
**AhRcomp (n=163)**				
∑ PCB_14	0.25	-0.04 (0.05), 0.34	0.93	-0.01**
∑ pesticide	0.23	-0.05 (0.04), 0.26	0.94	-0.01**
∑ POP	0.24	-0.05 (0.05), 0.30	0.93	-0.01**
**Across sexes (all districts)**				
**AhR-TEQ (n=254)**				
∑ PCB_14	0.13	**-0.08 (0.04), 0.05**	0.33	0.02
∑ pesticide	0.11	**-0.10 (0.04), 0.01**	0.47	0.03
∑ POP	0.13	**-0.1 (0.04), 0.02**	0.35	0.03
**AhRcomp (n=327)**				
∑ PCB_14	0.27	**-0.07 (0.02), 0.006**	**0.001**	0.08
∑ pesticide	0.31	**-0.07 (0.02), 0.003**	**0.001**	0.08
∑ POP	0.28	**-0.07 (0.02), 0.004**	**0.001**	0.08

Both AhR activities and POPs are ln transformed. Homogeneity of slope: test for homogeneity of association between exposure variables and outcome variables across the study group ($p > 0.05$, accept the hypotheses of homogeneity of slope). Common slope: the estimated common slope across study groups assuming homogeneity ($p > 0.05$, accept the hypotheses that slope equals to zero). Common intercept: test of a common intercept across study groups assuming a common slope ($p > 0.05$, accept the hypotheses having common intercept across the study groups). R^2 (Adjusted R square) assumes a common slope. Bold values are those showing significance.

*: Since heterogeneity of slope exists between POPs and AhRcomp across the districts, i.e. there were district differences in the associations of POP and AhRcomp, no further evaluation was performed.

**: equals to zero
